# COVID-19-Associated Myocarditis Managed With High-Dose Steroids: A Case of Rapid Clinical and Biochemical Recovery

**DOI:** 10.7759/cureus.95677

**Published:** 2025-10-29

**Authors:** Abhishek Pant, Shruti Gupta, Sachin Sapkota, Suchita Acharya, Patrick Kicker, Barney Soskin, Lela Ruck

**Affiliations:** 1 Internal Medicine, The Hospitals of Providence-Transmountain/Texas Tech University Health Sciences Center El Paso, Paul L. Foster School of Medicine, El Paso, USA; 2 Pulmonary and Critical Care Medicine, The Hospitals of Providence-Transmountain/Texas Tech University Health Sciences Center El Paso, Paul L. Foster School of Medicine, El Paso, USA

**Keywords:** covid-19, high-dose, methylprednisolone, myocarditis, steroids

## Abstract

Myocarditis is an uncommon but serious complication of SARS-CoV-2 infection. Its diagnosis remains challenging due to clinical features overlapping with acute coronary syndromes and the lack of standardized criteria; yet, early recognition is crucial to prevent fulminant progression. We report a case of a 74-year-old woman with a recent COVID-19 infection presenting with chest discomfort, new-onset atrial fibrillation, and rising troponin levels. Despite initial treatment for non-ST elevation myocardial infarction, she experienced worsening respiratory symptoms and chest pain. Workup, including echocardiography and left heart catheterization, excluded obstructive coronary disease and revealed inferior wall hypokinesis with preserved ejection fraction. Elevated inflammatory markers (C-reactive protein (CRP), interleukin-6 (IL-6)) and clinical deterioration supported the diagnosis of COVID-19-associated myocarditis. Initiation of high-dose intravenous methylprednisolone (500 mg daily for 3 days) resulted in rapid clinical and biochemical improvement. She was discharged on a tapering dose of steroids. Six weeks of follow-up showed resolution of myocarditis with normal troponin. While evidence for steroid use in viral myocarditis is mixed, emerging case reports and reviews support immunosuppressive therapy in COVID-19-associated myocarditis. This case highlights the therapeutic benefit of high-dose corticosteroids, likely by mitigating cytokine-mediated myocardial injury. COVID-19-associated myocarditis should be considered in patients with unexplained cardiac injury and recent SARS-CoV-2 infection. Our case demonstrates that the timely administration of high-dose corticosteroids may lead to favorable outcomes. Further research is warranted to establish standardized treatment protocols and validate the efficacy of immunosuppressive therapy in this population.

## Introduction

Myocarditis related to COVID-19 is an uncommon but very serious and life-threatening complication of SARS-CoV-2 infection. Myocardial damage resembling myocarditis was among the first complications reported in COVID-19 patients [1,2]. The American College of Cardiology (ACC) notes that the true incidence of myocarditis in the context of COVID-19 is challenging to determine due to varying definitions and study methodologies [3]. Centers for Disease Control and Prevention estimated COVID-19-related myocarditis at 150 per 100,000, versus 9 per 100,000 in non-COVID cases [4]. Rubens et al. analyzed the California State Inpatient Database data and found that myocarditis was linked to significantly higher in-hospital mortality (30.0% vs. 17.5%) [5]. Thus, early management of COVID-19 myocarditis is essential, preventing rapid progression to severe cardiac complications, including fulminant myocarditis. The American College of Cardiology's 2022 guidelines for COVID-19 myocarditis advocate for a personalized approach depending on the severity of the condition, with the use of corticosteroids and other supportive treatments [6]. Here, we present a case of a 74-year-old female with COVID-associated myocarditis who demonstrated significant clinical and biochemical improvement with high-dose steroids. This case report has been reported in line with the SCARE (Surgical CAse REport) 2025 criteria [7].

## Case presentation

Our case involves a 74-year-old female with a history of hypertension, hyperlipidemia, deep vein thrombosis, and transcatheter aortic valve replacement who presented with an acute onset of left arm numbness, left jaw pain, and a burning epigastric sensation. She denied dyspnea, cough, or palpitations. She had recently tested positive for SARS-CoV-2 four days prior and had completed a three-day course of Nirmatrelvir/Ritonavir.

**Figure 1:**
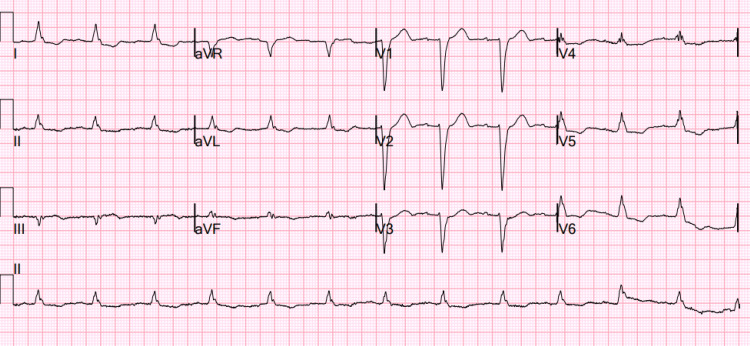
Electrocardiogram obtained in the emergency department following the administration of intravenous diltiazem Electrocardiogram showing sinus rhythm with a heart rate of 75 and a prolonged PR interval (211 mm), indicating a first-degree heart block along with a left bundle branch block (after the diltiazem dose)*.*

**Figure 2:**
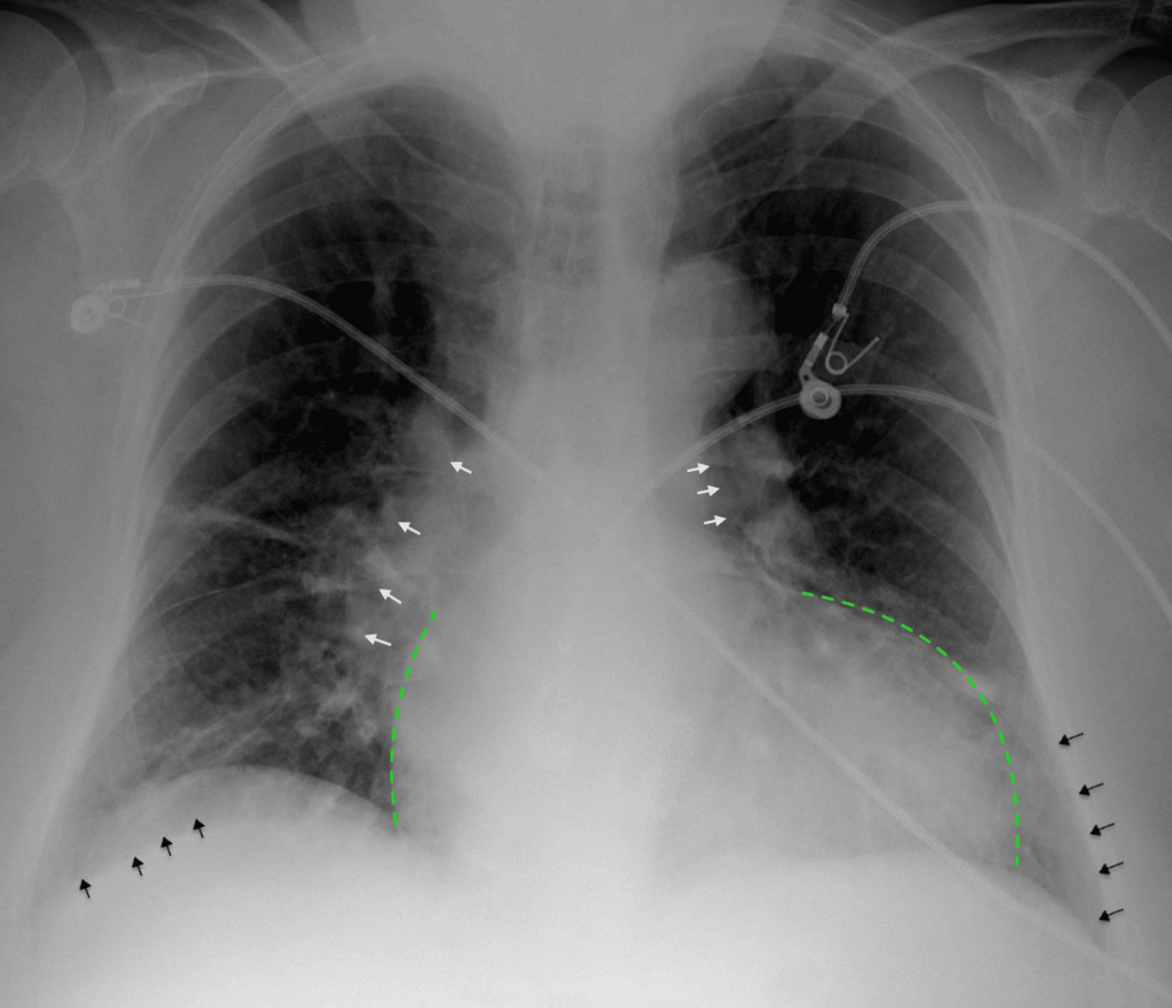
Chest X-ray (CXR) on admission Chest X-ray on admission demonstrating prominent central pulmonary vasculature (white arrows), linear atelectasis and/or scarring in the bilateral lower lung fields (black arrows), and cardiomegaly (green dotted lines).

**Figure 3:**
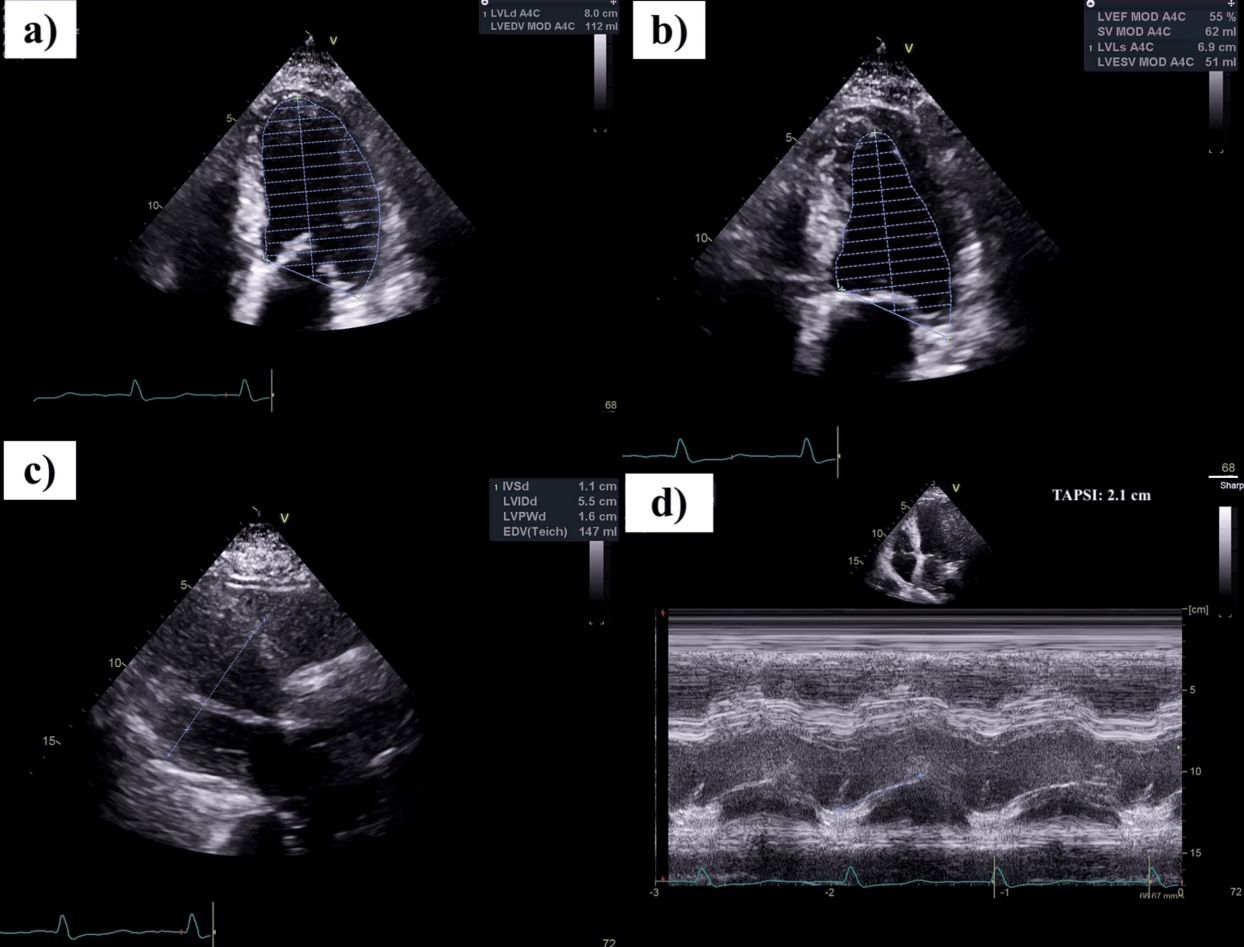
Echocardiogram on admission showing mild left ventricular hypertrophy and inferior wall hypokinesis Panels a and b: Left ventricular volumes in diastole (112 mL) and systole (51 mL), demonstrating an ejection fraction of 55%. The inferior wall appears hypokinetic.Panel c: Mild left ventricular hypertrophy (diastole) with an interventricular septal thickness of 1.1 cm and posterior wall thickness of 1.6 cm. Panel d: Tricuspid annular plane systolic excursion (TAPSE) measuring 2.1 cm, consistent with normal right ventricular systolic function.

On arrival, the patient was stable with normal oxygenation in room air. The exam showed clear lungs, regular heart sounds, and mild bilateral ankle edema. Laboratory evaluation revealed an elevated and up-trending troponin level (initially 82 ng/L rising to >26,000 ng/L), while complete blood count (CBC), comprehensive metabolic panel (CMP), and brain natriuretic peptide (BNP) were within normal limits (Table 1). C-reactive protein (CRP) and interleukin-6 (IL-6) were elevated. The initial electrocardiogram demonstrated sinus rhythm; however, a subsequent tracing revealed atrial fibrillation with a rapid ventricular response, for which a 20 mg intravenous dose of diltiazem was administered. The rhythm subsequently reverted spontaneously to sinus rhythm (Figure 1). Chest X-ray (CXR) showed prominent central pulmonary vasculature, linear atelectasis, and/or scarring in the lower lung fields bilaterally and cardiomegaly (Figure 2). Echocardiogram showed mild left ventricular hypertrophy, normal size and function (left ventricular ejection fraction (LVEF)​​ 50-55%), and inferior wall hypokinesis (Figure 3). The patient was admitted to the telemetry floors for further evaluation and management.

**Table 1:** Significant labs at admission and consecutive trends during hospitalization The table shows blood work trends with a marked rise in troponin and CRP levels, followed by a decline after the initiation of steroid therapy. The IL-6 level is also noted to be significantly elevated. WBC, white blood cell count; BNP, brain natriuretic peptide; CRP, C-reactive protein; CK, creatine kinase; LDH, lactate dehydrogenase; IL-6, interleukin-6

Parameter	Reference Range	Day 1 (Admission)	Day 3 (High-Dose Steroid initiation)	Day 5	Day 8 (Discharged)	Day 45 (Post-Steroid)
WBC	4.5-11	6.5	21.39	17.89	9.41	6.13
Hemoglobin	12-16	15.1	15.4	14.9	14.1	12.2
Platelets	130-400	249	224	287	267	240
Creatinine	0.3-1.3	0.8	1.0	1.2	0.8	0.8
BNP	<100 pg/ml	38	–	–	–	1031 >555
Troponin I	8.7-18.7 (pg/ml)	82.7	>26214	>26214	13480	35.3
CRP	0.10-1.0 (mg/dl)	–	23.16	14.92	4.15	–
CK	26-140 (IU/L)	2160	366	–	–	–
LDH	100-225 (U/L)	1366	–	–	–	–
IL-6	0.0-13.0 (pg/ml)	–	334	–	–	–

Suspected COVID-19-related myocardial infarction (MI) was treated with Aspirin, Clopidogrel, Atorvastatin, and Heparin. Despite non-ST-segment elevated myocardial infarction (NSTEMI) treatment, the patient developed worsening pleuritic chest pain and acute hypoxic respiratory failure (AHRF), requiring ICU transfer and high-flow oxygen. Troponin peaked at 26,214 ng/L. Given the persistent chest discomfort and biomarker trend, the patient underwent an urgent left heart catheterization, which ruled out obstructive coronary disease, showing only mild diffuse atherosclerosis and normal LV end-diastolic pressure. These findings, in conjunction with recent COVID-19 infection, elevated troponin, and echocardiographic evidence of inferior wall hypokinesis with preserved ejection fraction, directed the diagnosis toward COVID-19-associated myocarditis.

**Figure 4:**
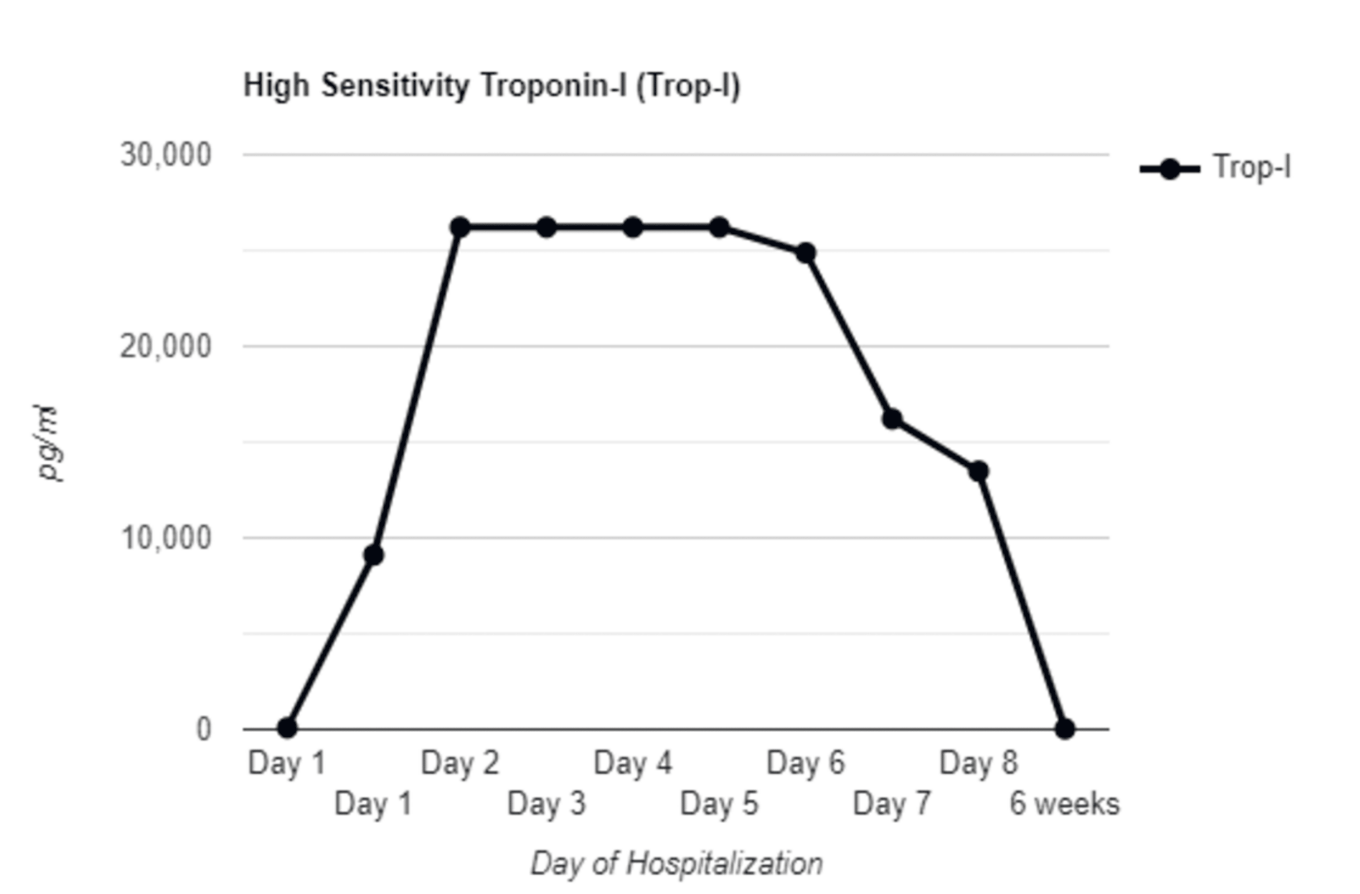
Trend of cardiac enzyme marker troponin-I over the course of hospitalization and the six-week follow-up The figure shows a sharp rise in troponin levels due to myocarditis, followed by a rapid decline after initiation of high-dose steroids during hospitalization, with sustained improvement at the six-week follow-up.

**Figure 5:**
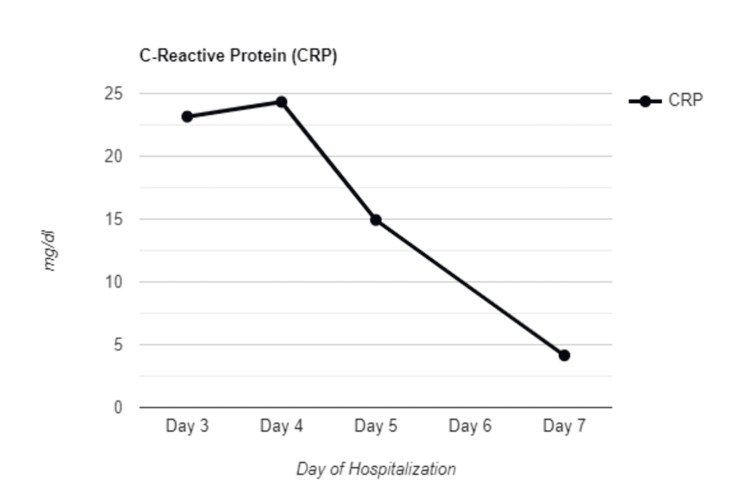
Trend of inflammatory marker C-reactive protein over the course of hospitalization The figure shows an initial rise in C-reactive protein levels due to myocarditis, followed by a rapid decline after the initiation of high-dose steroids during hospitalization.

For COVID-19 pneumonia, a 5-day course of Remdesivir (200 mg IV loading dose, then 100 mg daily) and Dexamethasone 6 mg daily was initiated. Given the high suspicion of COVID associated with myocarditis and lack of improvement with the above treatment, the patient was started on high-dose IV methylprednisolone at 500 mg per day, in divided doses, for a total of 3 days. Over the following days, the patient demonstrated clinical improvement with down-trending troponin (Figure 4) and CRP levels (Figure 5), improved oxygenation, and resolution of pleuritic chest discomfort. She was successfully weaned to a nasal cannula and ultimately discharged home on supplemental oxygen with exertion. A six-week oral Prednisone taper was prescribed along with guideline-directed medical therapy, including Metoprolol and Sacubitril/Valsartan. Outpatient follow-up with cardiology and cardiac MRI was advised. In week six, the patient presented to the emergency department with symptoms of congestive heart failure (CHF) exacerbation, attributed to one to two weeks of medication non-compliance. BNP was elevated on presentation but showed a down-trending pattern with appropriate heart failure management. However, troponin I levels remained within normal limits, indicating an improvement of COVID-associated myocarditis with corticosteroid therapy. The timeline of the events is presented in Table 2.

**Table 2:** Timeline of events COVID-19, coronavirus disease 2019; NSTEMI, non–ST-elevation myocardial infarction; AF, atrial fibrillation; RVR, rapid ventricular response; ICU, intensive care unit; LHC, left heart catheterization; CAD, coronary artery disease; IV, intravenous; CRP, C-reactive protein; GDMT, guideline-directed medical therapy; O₂, oxygen

Day	Event
Day -4	COVID-19 positive; on oral Nirmatrelvir/ Ritonavir
Day 0	Admission; presented with chest pain and up-trending troponin
Day 1	Admitted for NSTEMI; (AF with RVR → resolved)
Day 2	ICU transfer for acute hypoxic respiratory failure and severely elevated troponin; started on high-flow oxygen; started on Remdesivir and standard-dose steroids
Day 3	LHC: No CAD; diagnosis of COVID-19 myocarditis--> high-dose IV steroids started (Methylprednisone 500 mg daily)
Day 7	Symptoms improved, CRP normalized, troponin down-trended
Day 8	Discharged on steroid taper and GDMT
Week 6	Troponin normalized

Patient’s perspective

The patient was satisfied with the treatment and is doing well during outpatient follow-up at three months.

## Discussion

Although COVID-19 is primarily a respiratory illness, multiple reports have documented both acute and chronic cardiac complications associated with the inflammatory response. Myocarditis is one of the most frequently diagnosed cardiac manifestations in hospitalized patients with COVID-19. A nationwide Inpatient Sample Database reported a prevalence of 0.21% of myocarditis among 1,678,995 hospitalized COVID-19 patients [8].

Early detection and prompt treatment of myocarditis help minimize the risk of complications; however, there are no established guidelines for the pharmacological management of COVID-19-associated myocarditis. Lim et al. performed a systematic review of 146 case reports on COVID-19-associated myocarditis, where 84% recovered successfully, with most patients receiving steroid treatment (47.3%) [9]. A significant proportion of cases exhibited elevated troponin and CRP levels [9]. Elevated IL-6 and troponin have been strongly and independently associated with increased severity and in-hospital mortality [4]. Similar alarming labs with elevated CRP, Troponin, and IL-6 were observed in our case. The diagnosis of myocarditis in patients with COVID-19, in general, relied more heavily on clinical symptoms and the presence of elevated troponin without evidence of coronary artery disease, especially in the United States [4]. However, endo-myocardial biopsy (EMB), or direct histopathological examination, remains the gold standard for diagnosis of myocarditis [10,11]. Our patient's presentation was most consistent with COVID-19 myocarditis.

In non-COVID-19 myocarditis, evidence regarding the use of corticosteroids and immunosuppressive therapy is mixed, with the most significant benefit observed in specific myocarditis subtypes, including giant cell, eosinophilic, and non-viral myocarditis [12,13]. One of the systematic reviews that included 8 RCTs (with 719 participants) demonstrated a lack of mortality benefits with corticosteroids in patients with viral lymphocytic myocarditis and low ejection fraction [14]. Despite this, corticosteroids are often used in the acute phase of fulminant or hemodynamically compromised viral myocarditis. A systematic review by Sawalha et al. demonstrated favorable outcomes related to myocarditis treated with steroid therapy [15]. Another systematic review by Kamarullah et al. also reported that of 18 patients who received corticosteroid treatment, 13 (72%) showed significant clinical improvements during follow-up, while the remaining 5 (28%) experienced uneventful events [16]. Tocilizumab, an IL-6 inhibitor, was granted authorization by the US Food and Drug Administration for severe COVID-19 patients already on corticosteroids, but its effectiveness remains unclear [17]. Additionally, multiple case reports showed immunosuppression, including steroids, being an effective treatment strategy for COVID-associated myocarditis [18-20].

Our case shows rapid reversal of COVID-19-related cardiac injury after immunosuppressive treatment, with declining inflammatory markers, supporting cytokine storm as a cause. There are no standard guidelines regarding the dose, duration, or choice of steroid, making it dependent on the physician's judgment. This case advocates for further investigation to better define the role of immunosuppression in COVID-19-associated myocarditis.

## Conclusions

Myocarditis is a rare complication that requires early clinical suspicion for diagnosis and treatment. In patients with COVID-19, signs of cardiac injury, such as ECG changes or elevated troponin levels, should prompt clinicians to consider the possibility of concurrent myocarditis. Our case highlights the importance of the early identification and treatment of suspected myocarditis with high-dose intravenous corticosteroids, which played a key role in our patient’s full recovery. While systematic reviews support the use of corticosteroids in COVID-19-associated myocarditis, no randomized clinical trials have been conducted to evaluate the efficacy and safety of corticosteroids for treating myocarditis. Therefore, further well-designed randomized clinical trials are needed to validate these findings.

## References

[1] Huang C, Wang Y, Li X (2020). Clinical features of patients infected with 2019 novel coronavirus in Wuhan, China. Lancet.

[2] Wang D, Hu B, Hu C (2020). Clinical characteristics of 138 hospitalized patients with 2019 novel coronavirus-infected pneumonia in Wuhan, China. JAMA.

[3] Fairweather D, Beetler DJ, Di Florio DN, Musigk N, Heidecker B, Cooper LT Jr (2023). COVID-19, myocarditis and pericarditis. Circ Res.

[4] Boehmer TK, Kompaniyets L, Lavery AM (2021). Association between COVID-19 and myocarditis using hospital-based administrative data - United States, March 2020-January 2021. MMWR Morb Mortal Wkly Rep.

[5] Rubens M, Ramamoorthy V, Saxena A (2022). Hospital outcomes among COVID-19 hospitalizations with myocarditis from the California State Inpatient Database. Am J Cardiol.

[6] Gluckman TJ, Bhave NM, Allen LA (2022). 2022 ACC expert consensus decision pathway on cardiovascular sequelae of COVID-19 in adults: myocarditis and other myocardial involvement, post-acute sequelae of SARS-CoV-2 infection, and return to play: a report of the American College of Cardiology Solution Set Oversight Committee. J Am Coll Cardiol.

[7] Kerwan A, Al-Jabir A, Mathew G (2025). Revised Surgical CAse REport (SCARE) guideline: an update for the age of artificial intelligence. Premier Journal of Science.

[8] Sattar Y, Sandhyavenu H, Patel N (2023). In-hospital outcomes of COVID-19 associated myocarditis (from a nationwide Inpatient Sample database study). Am J Cardiol.

[9] Lim V, Topiwala G, Apinova E, Diioia M (2024). Systematic review of case reports on COVID-19 associated myocarditis: a discussion on treatments. Virol J.

[10] Cooper LT, Baughman KL, Feldman AM (2007). The role of endomyocardial biopsy in the management of cardiovascular disease: a scientific statement from the American Heart Association, the American College of Cardiology, and the European Society of Cardiology. Endorsed by the Heart Failure Society of America and the Heart Failure Association of the European Society of Cardiology. J Am Coll Cardiol.

[11] Howlett JG, Crespo-Leiro MG (2022). The International endomyocardial biopsy position paper: a basis for integration into modern clinical practice. J Card Fail.

[12] Frustaci A, Russo MA, Chimenti C (2009). Randomized study on the efficacy of immunosuppressive therapy in patients with virus-negative inflammatory cardiomyopathy: the TIMIC study. Eur Heart J.

[13] Cooper LT Jr, Berry GJ, Shabetai R (1997). Idiopathic giant-cell myocarditis--natural history and treatment. N Engl J Med.

[14] Chen HS, Wang W, Wu SN, Liu JP (2013). Corticosteroids for viral myocarditis. Cochrane Database Syst Rev.

[15] Sawalha K, Abozenah M, Kadado AJ (2021). Systematic review of COVID-19 related myocarditis: insights on management and outcome. Cardiovasc Revasc Med.

[16] Kamarullah W, Nurcahyani Nurcahyani, Mary Josephine C, Bill Multazam R, Ghaezany Nawing A, Dharma S (2021). Corticosteroid therapy in management of myocarditis associated with COVID-19; a systematic review of current evidence. Arch Acad Emerg Med.

[17] Lovell JP, Čiháková D, Gilotra NA (2022). COVID-19 and myocarditis: review of clinical presentations, pathogenesis and management. Heart Int.

[18] Hu H, Ma F, Wei X, Fang Y (2021). Coronavirus fulminant myocarditis treated with glucocorticoid and human immunoglobulin. Eur Heart J.

[19] Li A, Garcia-Bengochea Y, Stechel R, Azari BM (2020). Management of COVID-19 myopericarditis with reversal of cardiac dysfunction after blunting of cytokine storm: a case report. Eur Heart J Case Rep.

[20] Shabbir A, Camm CF, Elkington A, Tilling L, Stirrup J, Chan A, Bull S (2020). Myopericarditis and myositis in a patient with COVID-19: a case report. Eur Heart J Case Rep.

